# Light, chromatin, action: nuclear events regulating light signaling in Arabidopsis

**DOI:** 10.1111/nph.18424

**Published:** 2022-09-07

**Authors:** Eirini Patitaki, Geoffrey Schivre, Anna Zioutopoulou, Giorgio Perrella, Clara Bourbousse, Fredy Barneche, Eirini Kaiserli

**Affiliations:** ^1^ School of Molecular Biosciences, College of Medical, Veterinary and Life Sciences University of Glasgow Glasgow G12 8QQ UK; ^2^ Institut de Biologie de l'École Normale Supérieure (IBENS), École Normale Supérieure, CNRS, INSERM Université PSL Paris 75005 France; ^3^ Université Paris‐Saclay Orsay 91400 France; ^4^ Department of Biosciences University of Milan Via Giovanni Celoria, 26 20133 Milan Italy

**Keywords:** chromatin, epigenetics, phase separation, photomorphogenesis, plant development, transcription

## Abstract

The plant nucleus provides a major hub for environmental signal integration at the chromatin level. Multiple light signaling pathways operate and exchange information by regulating a large repertoire of gene targets that shape plant responses to a changing environment. In addition to the established role of transcription factors in triggering photoregulated changes in gene expression, there are eminent reports on the significance of chromatin regulators and nuclear scaffold dynamics in promoting light‐induced plant responses. Here, we report and discuss recent advances in chromatin‐regulatory mechanisms modulating plant architecture and development in response to light, including the molecular and physiological roles of key modifications such as DNA, RNA and histone methylation, and/or acetylation. The significance of the formation of biomolecular condensates of key light signaling components is discussed and potential applications to agricultural practices overviewed.

**Table:** 

	Summary	333
I.	Introduction	333
II.	Light‐mediated regulation of transcription and its link to chromatin status	335
III.	Light‐driven regulation of histone composition	338
IV.	Light‐mediated regulation of chromatin architecture	339
V.	Light‐driven regulation of the epitranscriptome	342
VI.	An epigenetic perspective on light regulation of genome and epigenome dynamics	343
VII.	Conclusions and future directions	344
	Acknowledgements	345
	References	345

## I. Introduction

Sunlight is a pivotal environmental stimulus for autotrophic plants as it provides the ultimate energy source for photosynthesis, whilst light cues also direct morphological, architectural and physiological responses (Mayer, 1845; Franklin *et al*., 2005; Kami *et al*., 2010). As sessile organisms, flowering plants have developed sophisticated molecular mechanisms to perceive and adapt to changes in light conditions, which ensure survival and reproductive success. Light‐driven plant physiological adaptations and developmental transitions include seed germination, photomorphogenesis (or de‐etiolation) and flowering initiation, whereas short‐term processes such as circadian clock entrainment, phototropism, shade avoidance or stomatal aperture and chloroplast movements are influenced by light signaling to anticipate or adjust plant capacity to cope with a changing environment. Although suboptimal light energy or wavelengths can affect the plant energetic status, extreme light intensities can induce several types of damage to proteins and DNA with multiple consequences ranging from plastid activity to genome stability. Moderate‐to‐high intensities of UV‐B irradiation can cause DNA damage in the form of photo‐adducts and the production of reactive oxygen species (ROS) that can lead to a reduction in photosynthetic yield and ultimately cell death (Britt, 1995; Frohnmeyer & Staiger, 2003; Favory *et al*., 2009; Shi & Liu, 2021). In addition, prolonged exposure to high light intensity can lead to energy profuse that exceeds the photosynthetic capacity of plants (Mishra *et al*., 2012). A decrease in photosynthetically active radiation (PAR) or a reduction in the red to far‐red ratio (R : FR) induced by plant proximity or canopy shade can also trigger adaptive responses in shade‐avoidant species, such as *Arabidopsis thaliana*. Shade avoidance response (SAR) is characterized by leaf hyponasty, hypocotyl and leaf elongation, and early flowering initiation to enhance light‐harvesting or temporally overcome competing vegetation by enhancing reproductive success (Morgan & Smith, 1978; Smith, 1982; Smith & Whitelam, 1997).

Plants sense diurnal and seasonal as well as unpredictable changes in light properties through a complex photosensory system that relies on photoreceptor proteins (Smith, 1982; Briggs & Olney, 2001; Paik & Huq, 2019). Vascular plants utilize five families of photoreceptors that perceive different spectrum wavelengths, depending on their biochemical properties. Phytochromes (phyA–phyE) are activated by R and FR light; cryptochromes (CRY1, CRY2 and CRY3), phototropins (phot1 and phot2) and F‐box containing Flavin binding proteins (ZEITLUPE (ZTL) and FLAVIN‐BINDING, KELCH REPEAT, F‐BOX 1/LOV KELCH PROTEIN 2 (FKF1/LKP2)) absorb UV‐A and blue light, whereas UVR8 (UV‐RESISTANCE LOCUS 8) perceives UV‐B and UV‐A light (Sharrock & Quail, 1989; Clack *et al*., 1994; Lin *et al*., 1996; Rizzini *et al*., 2011; Christie *et al*., 2012, 2015). Upon photoexcitation, photoreceptors undergo structural changes and transit to the activated state which grants the initiation of light signal transduction (Harper *et al*., 2003; Kami *et al*., 2010). Although photoreceptor families differ in structure, they can trigger downstream signaling through a series of molecular signal transduction events that constantly regulate the plant transcriptome. Genomic studies estimate that minimally 30% of the Arabidopsis transcriptome is modulated during photomorphogenesis. Transcriptional regulation is the cornerstone of photomorphogenesis and is largely controlled by a small number of transcription factors (TFs) including the master regulator ELONGATED HYPOCOTYL 5 (HY5) and a family of PHYTOCHROME INTERACTING FACTORs (PIFs), each targeting hundreds of genes involved in multiple light‐regulated pathways (Jiao *et al*., 2007; Perrella & Kaiserli, 2016; Bourbousse *et al*., 2020). Furthermore, epigenome modifiers typically classified as ATP‐dependent chromatin remodelers, histone chaperones or histone‐modifying enzymes acting as writers or erasers can function independently or together with transcriptional regulators to shape the epigenome landscape in response to fluctuating environmental conditions altogether influencing transcription and chromatin architecture (Berger, 2007; Pikaard & Scheid, 2014).

The elemental unit of chromatin, the nucleosome, is organized as a histone octamer made of two copies of each core histone H2A, H2B, H3 and H4 around which 146 bp of DNA are wrapped and can be further compacted by the linker histone H1 (Kouzarides, 2007). Both histone tails and core domains are enriched in basic amino acids, like lysine (K) and arginine (R), which can be reversely modified by the addition and/or removal of different chemical components that alter DNA accessibility and/or attract *trans* factors. During DNA replication or in response to specific signals including environmental stimuli, nucleosomes can also incorporate histone variants such as H2A.Z and H3.3 that impact on chromatin chemico‐physical properties at specific chromatin regions (Wollmann *et al*., 2017; Lei & Berger, 2020; Bieluszewski *et al*., 2022). Myriads of histone post‐translational modifications (PTMs) further contribute to adjusting the chromatin status along the genome. Chromatin marks have long been thought to define a so‐called histone code superimposing with the genetic code to regulate most, if not all, cellular functions (Berger, 2007).

DNA methylation is another central regulatory mechanism playing a pivotal role in gene expression, genome stability and epigenetic processes (Zhang *et al*., 2018). In Arabidopsis, the DNA methylation machinery can target cytosines (C) in any sequence context (CG, CHG and CHH; Martienssen & Colot, 2001). DNA methylation is particularly abundant at DNA repeats such as silent transposable elements (TEs) and other genome scaffolding domains such as ribosomal RNA genes where chromatin is highly compacted, poorly accessible to the transcriptional machinery, and associated to silencing factors (Ichino *et al*., 2021). Enrichment of methylation at cytosines (mCG) is also found within the transcribed regions of long and slowly evolving genes that tend to show stable expression across tissues and conditions (Bewick & Schmitz, 2017). Cytosine methylation can be established *de novo* by RNA‐directed DNA Methylation (RdDM), which begins with the generation of small RNAs and ends with the methylation of cytosines in all sequence contexts CG, CHG and CHH by the DNA methyltransferase DRM2 (DOMAINS REARRANGED METHYLTRANSFERASE 2; Law & Jacobsen, 2010; Matzke *et al*., 2015; To & Kakutani, 2022). DNA methylation then can be maintained by other methyltransferases such as MET1 (METHYLTRANSFERASE 1) mediating CG methylation, while CMT3 (CHROMOMETHYLASE 3) operates in CHG methylation, and DRM1 and 2 (DOMAINS REARRANGED METHYLTRANSFERASE 1 and 2) methylate non‐CG sites (Vanyushin & Ashapkin, 2011; Pikaard & Scheid, 2014). Combined dynamic modulation of histone and DNA composition and organization regulate genome compartmentalization between euchromatin (gene‐rich and usually accessible to the transcriptional machinery) and heterochromatin (repeat‐rich, highly condensed and transcriptionally silent; Riddle *et al*., 2011).

In addition to chromatin and DNA modifications, post‐transcriptional RNA modifications also contribute to the regulation of the plant transcriptome (Liang *et al*., 2020) and therefore can be considered as being part of the epigenetic system. So far, N6‐methyladenosine (m^6^A) and 5‐methylcytosine (m^5^C) have been detected in Arabidopsis messenger RNA (mRNA) and affect mRNA stability, interactions with other molecules, as well as secondary structure (Chmielowska‐Bąk *et al*., 2019). Eminent reports also suggest that mRNA modifications play an important role in RNA metabolism including transcript processing, translational efficiency, splicing, decay and transport (Zhao *et al*., 2017; Kadumuri & Janga, 2018; L. Shen *et al*., 2019). Recent epitranscriptome studies also hint at their involvement in many plant physiological processes such as root and trichome development, flowering and leaf initiation, shoot stem cell fate and embryo development (L. Shen *et al*., 2019).

**Fig. 1:**
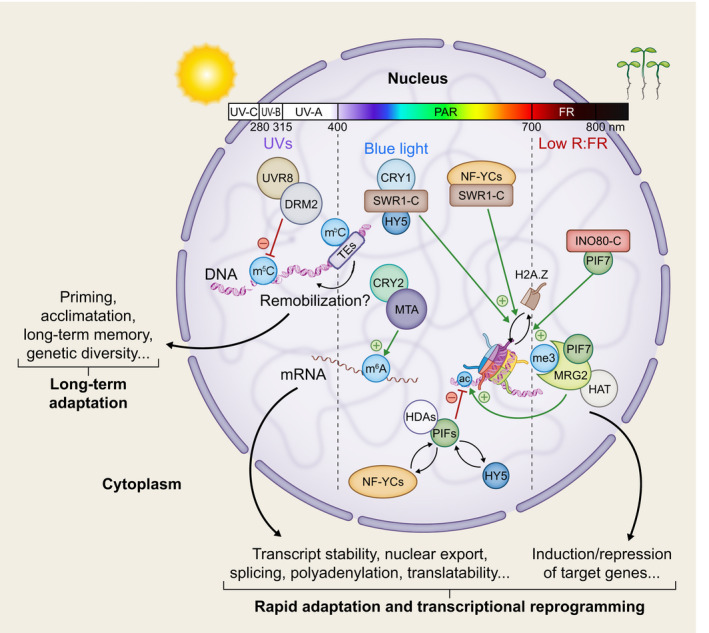
Epigenetic and epitranscriptomic signaling converge to equip plants with adaptive strategies in response to changing light environments. UV‐B light triggers monomerization and nuclear import of UVR8 which then interacts and inhibits the DNA methyltransferase activity of DRM2 (Jiang *et al*., 2021). The global UV‐B induced DNA hypomethylation could provide grounds for long‐term plant adaptation through epigenetic memory or remobilization of transposable elements leading to genetic diversification. Core light signaling components physically interact with chromatin modifiers and remodelers (see Table 1 for details and references) to fine‐tune the expression of light‐responsive genes. HDAs are, for example, alternatively recruited by PIFs, NF‐YCs or HY5 leading to repression of target genes associated with responses such as auxin transport or cell wall loosening. Conversely, upon low red to far‐red light ratio (low R : FR), MRG2 interacts with PIF7, recognizes H3K4me3 or H3K36me3 marks and recruits an unknown HAT to activate the expression of shade responsive genes promoting hypocotyl elongation (Peng *et al*., 2018). Incorporation of histone variants such as H2A.Z is also a common route to convey light signaling as exemplified under shade in which PIF7 recruits the INO80 complex (INO80‐C) to target genes, removing H2A.Z from gene body and promoting their transcription (Willige *et al*., 2021). H2A.Z also is involved in blue light signaling via SWI2/SNF2‐RELATED 1 chromatin remodeling complex (SWR1‐C) recruitment by CRY1 (see Fig. 2) or NF‐YCs to dampen the transcription of auxin‐responsive and cell wall‐loosening genes, thus slowing hypocotyl growth during photomorphogenic development. Finally, blue light signaling involves epitranscriptomic regulation through the recruitment of MTA by CRY2 affecting transcripts of circadian and other genes (Wang *et al*., 2021b). These mechanisms allow plants to modulate the expression of target genes providing ground for rapid adaptation to changing light conditions. UVR8, UV‐RESISTANCE LOCUS 8; DRM2, DOMAINS REARRANGED METHYLTRANSFERASE 2; HDA, HISTONE DEACETYLASE; NF‐YC, NUCLEAR FACTOR‐Y Subunit C; PIF7, PHYTOCHROME INTERACTING FACTOR 7; CRY, CRYPTOCHROME; MTA, mRNA ADENOSINE METHYLASE.

In this review, we report key advances in the areas of chromatin‐level regulation of light responses in Arabidopsis with a focus on the role of DNA, RNA and protein localization in shaping the nuclear landscape and triggering adaptive responses to changing light regimes (Fig.1). Open questions and insights on deciphering the mechanism underlying this regulation are highlighted and possible avenues for applications in agriculture are discussed.

## II. Light‐mediated regulation of transcription and its link to chromatin status

Early studies linking histone acetylation, nucleosome occupancy and transcription rate when comparing green and etiolated plant extracts indicate a general role for chromatin‐based mechanisms in the control of light‐dependent gene expression (Chua *et al*., 2001, 2003; Offermann *et al*., 2006). Even though single‐cell information is currently lacking, organ‐specific analyses of nuclear (Bourbousse *et al*., 2015), transcriptome (Lopez‐Juez *et al*., 2008; Kohnen *et al*., 2016; Sun *et al*., 2016; Burko *et al*., 2020) and TF footprint dynamics (Sullivan *et al*., 2014) provide evidence that spatio‐temporal chromatin regulation of gene expression in response to light is specifically achieved in different cell types in order to enable concerted physiological and developmental responses at the organismal level (He *et al*., 2011; Martínez‐García *et al*., 2014; Wu, 2014; Bourbousse *et al*., 2020; Jarad *et al*., 2020; Perrella *et al*., 2020; Tognacca *et al*., 2020). As represented in Table 1 and Fig. 1, an ever‐increasing number of studies has contributed to our knowledge on the signaling paths mediating direct or indirect regulation of gene expression in response to diverse light conditions.

**Table 1:** List of chromatin factors and light signaling transducers reported to physically interact.

Light signaling factor	Chromatin factor	Associated chromatin feature	Light response	Target genes	Biological processes	Publication
HY5 / HYH	PKL	Chromatin remodeling, H3K27me3	De‐etiolation	*DWF4*, *EXT3*, *XTH17*, *XTR6*, *EXP2* and *IAA19*	Hypocotyl elongation	Jing *et al*. (2013)
PIF3	HDA15	H4ac	De‐etiolation	*GUN5*, *PSBQ*, *LHCB2.2*, *PSAE1*	Chlorophyll biosynthesis & photosynthesis	Liu *et al*. (2013)
PIF1/3/4/5	DET1	H2BUb	De‐etiolation	Nd	Hypocotyl elongation	Dong *et al*. (2014)
PIF3	PKL	Chromatin remodeling	De‐etiolation	*IAA19*, *PRE1*	Hypocotyl elongation	Zhang *et al*. (2014)
NF‐YC1/3/4/9	HDA15	H4ac	De‐etiolation	*IAA19*, *XTH17*	Hypocotyl elongation	Tang *et al*. (2017)
PIF1	HDA15	H3ac	Germination	PINs, XTHs, EXPs	Hypocotyl elongation	Gu *et al*. (2017)
PIF7	MRG2	H3K4me3, H3K36me3 H4K5ac, H3K9ac, H3K14ac, H3K27ac, H3K36ac	SAS	*YUCCA8*, *IAA19*, *PRE1*	Hypocotyl elongation	Peng *et al*. (2018)
CO	PKL / ATX1	Chromatin remodeling, H3K27me3, H3K4me3	Flowering	*FT*	Flowering Time	Jing *et al*. (2019)
HY5	HDA15	H4ac	De‐etiolation	XTHs, *BXL1*, *EXP2*, PMEs	Hypocotyl elongation	Zhao *et al*. (2019)
HY5	HDA9	H3K9ac, H3K27ac	De‐etiolation	*ATG5*, *ATG8e*	Autophagy	Yang *et al*. (2020a)
HY5	HDA19	H3ac, H3K9ac	De‐etiolation	*HY5*, *BBX22*	Photomorphogenesis	Jing *et al*. (2020)
UVR8	DRM2	CHH DNA methylation	UV‐B	*Chr1:23068006*, *AT4TE29620*, *AT1TE55145*	DNA damage	Jiang *et al*. (2021)
PIF1	JMJ17	H3K4me3	De‐etiolation	*POR‐C*	Chlorophyll biosynthesis	Islam *et al*. (2021)
HY5 (+ CRY1)	ARP6 + SWC6 (SWR1‐C)	H2A.Z	De‐etiolation	*EXP2*, *IAA19*, *XTH33*	Hypocotyl elongation	Mao *et al*. (2021)
PIF7 (+ PIF4/5)	EEN (INO80)	H2A.Z, H3K9ac	SAS	*ATHB2* + *PIF7* targets (genome‐wide))	Hypocotyl elongation	Willige *et al*. (2021)
PhyB	VIL1 (PRC2)	H3K27me3	Red light	*ATHB2*, *HFR1*, *PIL1*	Hypocotyl elongation	Kim *et al*. (2021)
NF‐YC3/4/9	ARP6	H2A.Z	De‐etiolation	*IAA6*, *IAA19*	Hypocotyl elongation	C. Zhang *et al*. (2021)

Direct interactions between light signaling components and chromatin factors are listed with the chromatin features involved (either deposited/erased or recognized by the chromatin factor), the light conditions under which the interaction has been described, the target genes that have been monitored, the impacted biological process and the associated publications. Ac, acetylation; ARP6, ARP6, ACTIN‐RELATED PROTEIN 6; ATG, AUTOPHAGY; ATHB2, *ARABIDOPSIS THALIANA* HOMEOBOX PROTEIN 2; BBX, B‐BOX DOMAIN PROTEIN; BXL, BETA XYLOSIDASE; CO, CONSTANS; COP1, CONSTITUTIVE PHOTOMORPHOGENIC 1; CRY, CRYPTOCHROME; DRM2, DOMAINS REARRANGED METHYLTRANSFERASE 2; DWF, DWARF; EXT, EXTENSIN; DET1, DE‐ETIOLATED 1; EEN, EIN6 ENHANCER; EXP2, EXPANSIN 2; FT, FLOWERING LOCUS T; GUN, GENES UNCOUPLED; HDA, HISTONE DEACETYLASE; HFR1, LONG HYPOCOTYL IN FAR‐RED; H, HISTONE; HY5, ELONGATED HYPOCOTYL 5; HYH, ELONGATED HYPOCOTYL 5‐LIKE; IAA, INDOLE‐3 ACETIC ACID INDUCIBLE; JMJ, JUMONJI; LHCB, LIGHT HARVESTING COMPLEX; Me, methylation; MRG, MORF RELATED GENE; MTA, mRNA ADENOSINE METHYLASE; Nd, not determined; NF‐YC, NUCLEAR FACTOR‐Y Subunit C; PIF, PHYTOCHROME INTERACTING FACTOR; phy, PHYTOCHROME; PIL1, PHYTOCHROME INTERACTING FACTOR 3‐LIKE 1; PIN1, PIN‐FORMED 1; PKL, PICKLE; PME, PECTIN METHYLTRANSFERASE; PRC2, Polycomb Repressive Complex 2; PRE1, PACLOBUTRAZOL RESISTANCE 1; POR C, PROTOCHLOROPHYLLIDE OXIDOREDUCTASE C; PSAE, PHOTOSYSTEM I SUBUNIT E; PSBQ, PHOTOSYSTEM II SUBUNIT Q‐2; SWR1, SWI2/SNF2‐Related 1 Chromatin Remodeling Complex; SAS, Shade Avoidance Syndrome; Ub, ubiquitylation; UVR8, UV‐RESISTANCE LOCUS 8; VIL1, VERNALIZATION INSENSITIVE 3‐LIKE 1; XTH, XYLOGLUCAN TRANSFERASE; XTR, XYLOGLUCAN ENDOTRANSGLYCOSYLASE.

Although chromatin regulatory pathways typically act in a gene‐specific manner through the action of transcription factors, such as HY5, PIFs or NUCLEAR FACTOR‐Y (NF‐Y), by recruiting or driving chromatin components at specific loci (C. Zhang *et al*., 2021), several reports indicate that during light‐driven cellular transitions gene‐specific regulatory mechanisms are either integrated with higher order dynamics or collectively contribute to regulate the transcriptional regime. First, *in vitro* studies using nuclear extracts from Arabidopsis cultured cells suggested that chromatin constitutes a key determinant of light‐dependent transcriptional regulation, notably because four genes encoding Rubisco small subunits (rbcS‐1A, rbcS‐1B, rbcS‐2B and rbcS‐3B) showed no photodependent RNA Pol II (RNPII) activity when using naked DNA as a template, but did so when using reconstituted mammalian chromatin (Ido *et al*., 2016). Although this artificial experimental design using extracellular extracts may not be compared to living plant nuclei, several studies jointly shed light on global regulatory mechanisms that influence both the nucleus organization, the epigenome landscape, the RNPII transcriptional regime, and RNA synthesis and processing. Extending the gene‐specific transcriptional activation process initially observed by run‐on assays at the *PetE* photosynthetic gene promoter in green and etiolated shoots of pea seedlings (Chua *et al*., 2001), quantification of absolute and relative levels of RNPII active forms in individual nuclei unveiled that de‐etiolation is accompanied by > 2‐fold increase of transcription elongation activity per genome content in cotyledon cells (Bourbousse *et al*., 2015). Using a combination of RNPII chromatin immunoprecipitation (ChIP) and nascent RNA analyses for a subset of genes, recent work further showed that light can enhance RNPII processivity and thereby impact both RNA synthesis and splicing decisions (Herz *et al*., 2019).

Currently, we lag in understanding whether the activity of RNPII is directly regulated by light‐derived signals, for example by cyclin‐dependent kinases that phosphorylate its carboxy‐terminal domain (CTD), by RNPII‐associated Transcription Elongation Factors (Antosz *et al*., 2017), and/or by transcription coactivators (e.g. the PIF4‐associated MED25/PFT1 Mediator subunit; Cerdan & Chory, 2003; Klose *et al*., 2012; Sun *et al*., 2020). Nevertheless, several studies point to higher‐order chromatin dynamics as possible modulators of genome transcriptional competency. The latter possibility is supported first by occurrence of enormous changes in DNase Hypersensitive Sites (DHS) during Arabidopsis de‐etiolation (Sullivan *et al*., 2014), indicating that chromatin accessibility is strongly remodeled during dark‐to‐light transitions. Additionally, whereas light‐regulated chromatin footprints and accessibility are intimately linked to TF binding at multiple target genes (Sullivan *et al*., 2014), they also appear to be modulated by global changes in the abundance of multiple chromatin remodelers. For example, BAF60 (also named CHC1 or SWP73B) accumulates during dark‐to‐light transitions and is recruited to gene promoters where it antagonizes PIF4 activity through competitive binding onto G‐box motifs (Jegu *et al*., 2017). Reciprocally, the BRAHMA SWI2/SNF2‐type ATPase protein accumulates in dark conditions and physically associates with PIF1, mediating a *cis*‐regulatory gene repression mechanism of chlorophyll biosynthetic genes (Zhang *et al*., 2017). Lastly, the ATP‐dependent chromatin remodeling factor INOSITOL REQUIRING 80 (INO80) is degraded by the 26S proteasome pathway in the dark and accumulates in light conditions enabling chromatin incorporation of the H2A.Z histone variant at dark‐ and light‐induced genes where it presumably impacts transcription (Yang *et al*., 2020b).

Evidence of general adjustment of the epigenome to transcriptional competency by light is further supported by the large variations in the abundance of chromatin components, such as the linker histone variant H1.3 and the monoubiquitinated histone H2B (H2Bub) mark (Rutowicz *et al*., 2015; Nassrallah *et al*., 2018). H1.3 incorporation may trigger the formation of specific chromatin compaction states under unfavorable light conditions such as shade, whereas H2Bub enrichment over most transcribing genes during Arabidopsis de‐etiolation probably enhances RNPII transcriptional elongation (Bourbousse *et al*., 2012). In yeast and mammals, co‐transcriptional cycles of histone H2B mono‐ubiquitination, by E3 ubiquitin ligases and de‐ubiquitination by the SAGA complex, facilitates RNPII processivity across nucleosomes (Henry *et al*., 2003). H2Bub is typically associated with transcriptionally permissive chromatin in Arabidopsis as well in species in which H2Bub homeostasis along the genome is regulated by light signaling (Nassrallah *et al*., 2018). Regulation of H2Bub chromatin abundance by light is directly mediated by light signaling components, in particular DE‐ETIOLATED‐1 (DET1), a light signaling integrator (Chory *et al*., 1989) with a strong affinity for histone H2B (Benvenuto *et al*., 2002). As part of the C3D complex (comprising of COP10 (CONSTITUTIVE PHOTOMORPHOGENIC 10), DET1, DDB1 (DAMAGED DNA BINDING PROTEIN 1), and DDA1 (DDB1‐ASSOCIATED 1)), DET1 mediates ubiquitin‐mediated proteolytic degradation of a SAGA‐like de‐ubiquitination module (DUBm) in darkness, thereby regulating H2Bub levels over most, if not all, Arabidopsis genes (Nassrallah *et al*., 2018). Accordingly, H2Bub deposition acts in *cis* for efficient inducibility of hundreds of genes during Arabidopsis de‐etiolation, most notably long genes (> 4 kb) that may be particularly dependent on mechanisms facilitating RNPII processivity across nucleosomal physical barriers (Bourbousse *et al*., 2012). Likewise, RNA Polymerase II Associated Factor1 (PAF1) complex subunits, including EARLY FLOWERING7 (ELF7), are expressed at low levels in dark‐adapted Arabidopsis (Herz *et al*., 2019), possibly contributing also to the reduction of H2Bub levels and RNPII elongation capacity during plant adaptation to darkness.

## III. Light‐driven regulation of histone composition

### 1. Histone methylation

Histone methylation is regulated by the opposing activities of different histone methyltransferases (HMTs) and demethylases and recognized by several histone readers. Among the best studied histone post‐translational modifications (PTMs), lysine and arginine residues can be covalently mono‐, di‐, tri‐methylated at different positions along the histone H3 and H4 tails protruding out from the nucleosome core particle (e.g. H3 Lys‐K4, 9, 27 and 36 or H4 Lys‐20; Liu *et al*., 2010). Although the biochemical function of histone methylation remains elusive, the position and type of the modified residue is tightly linked to local transcriptional activation or repression. For example, in plants as in other eukaryotes H3K4me2/me3, H3K36me3 typically are associated with transcription, accumulating particularly around the transcriptional start site (TSS), whilst marks such as H3K9me2 are distributed along heterochromatin regions and the Polycomb Repressive Complex 2 (PRC2) chromatin hallmark H3K27me3 is usually correlated with gene repression (Ha *et al*., 2011).

Arabidopsis seedling de‐etiolation involves an increase in H3K4me3 at the TSS of *LIGHT HARVESTING COMPLEX* (*LHC*) *LHCB1.4*, *LHCB1.5*, *HCF173* (*HIGH CHLOROPHYLL FLUORESCENCE PHENOTYPE*) and *TZP* (*TANDEM ZINC‐FINGER PLUS3*) genes that correlates with their induction by light (Guo *et al*., 2008; Charron *et al*., 2009; Bourbousse *et al*., 2012) and contributes to efficient inducibility of such genes during the transition (Fiorucci *et al*., 2019). Among the many HMT activities, COMPASS (Complex Associated to Set 1) and the SET DOMAIN GROUP 2 (SDG2) trigger H3K4me3 deposition at several light‐inducible genes (Fiorucci *et al*., 2019) whereas SET DOMAIN GROUP 8 (SDG8) deposits H3K36me3 at light‐responsive elements (LREs; Li *et al*., 2015). *Vice versa*, a detailed case study of *PHYA* gene downregulation during Arabidopsis de‐etiolation identified dynamic erasure of H3K4me3 at the *PHYA* locus within 1 h of light exposure. Conversely, the *PHYA* locus depicts a quick increase of H3K27me3, thereby exemplifying the influence of light on chromatin state transitions through reversible histone modification (Jang *et al*., 2011).

Another phytochrome‐regulated response controlled by histone methylation is SAR. Phenotypic analysis of mutants for the histone methylation readers MORF RELATED GENE 1 (MRG1) and MRG2 display a significant reduction in hypocotyl elongation upon exposure to shade (Peng *et al*., 2018). Interestingly MRG2 can interact directly with PIF7 and together regulate the expression of shade‐responsive genes, including *YUCCA8* (*YUC8*), *YUCCA9* (*YUC9*), *PRE1* (*PACLOBUTRAZOL RESISTANCE 1*) and *IAA19* (*INDOLE‐3‐ACETIC ACID INDUCIBLE 19*). In addition, both MRG2 and PIF7 associate to modulate H3K4me3 and H3K36me3 distribution on LREs (such as the G‐box) and TSS regions on the aforementioned shade‐responsive genes (Peng *et al*., 2018).

Interestingly, the SUVH5 HMT acts as a positive regulator of phyB‐dependent seed germination (Gu *et al*., 2019). In particular, *suvh5* mutant seeds showed a reduction in the germination rate, under R light conditions, when phyB is most active (Gu *et al*., 2019). Whether phyB and SUVH5 function synergistically within the same pathway remains to be assessed. Conversely, the histone demethylases JUMONJI (JMJ) 20 and JMJ22 work together as positive regulators of seed germination in a phyB‐dependent manner (Cho *et al*., 2012). More specifically, upon light exposure phyB mediates the downregulation of the repressor SOMNUS via PIF‐LIKE5 (PIL5 or PIF1) protein degradation. SOMNUS inactivation allows expression of JMJ20/22 that removes H3R3me2 marks on *GIBBERELLIN 3‐BETA‐DIOXYGENASE* (*GA3OX*) 1 and *GA3OX2* loci, thereby triggering the accumulation of active GA in seeds essential for germination (Cho *et al*., 2012).

Light‐dependent developmental transitions are also mediated by the action of chromatin remodelers. PICKLE (PKL), an ATP‐dependent chromatin remodeling enzyme, was identified through a forward genetic screen as a negative regulator of de‐etiolation (Jing *et al*., 2013) that physically and genetically interacts with HY5, thereby modulating the expression of cell‐elongation related genes. HY5 recruits PKL to the *EXPANSIN2* and *IAA19* gene promoters, where PKL antagonizes HY5 action by reducing H3K27me3 levels. Altogether, this suggests the existence of a gene regulatory feedback loop modulating hypocotyl elongation (Jing *et al*., 2013). A similar mechanism also was identified during skotomorphogenesis, where PKL represses H3K27me3 deposition in response to brassinosteroid and gibberellin signaling (Zhang *et al*., 2014). PKL can also contribute to *FT* activation during photoperiodic flowering (Jing *et al*., 2019).

### 2. Histone acetylation

Histone acetylation is usually associated with an increase in gene expression, presumably because acetyl groups cause the neutralization of the chromatin charge, weaken DNA histone associations, and promote DNA accessibility to DNA effectors and to the transcriptional machinery (Jiang *et al*., 2020). Histone H3 and H4 present six (K9, K14, K18, K23, K27, K56) and five (K5, K8, K12, K16, K20) residues that can be acetylated, respectively (Hu *et al*., 2019). The modification of such residues is mediated by the antagonistic action of histone acetyl transferases (HATs) and histone deacetylases (HDACs; Pandey *et al*., 2002). In plants, HATs are grouped in four main families: GNAT (GCN5‐ related N‐terminal acetyltransferases), MYST, p300/CREB‐binding protein (CPB) and TATA binding protein‐associated factors (TAFs).

Early work on histone acetylation dynamics during Arabidopsis de‐etiolation unveiled that GCN5 represses hypocotyl elongation under FR light (Benhamed *et al*., 2006). In addition, HISTONE ACETYLTRANSFERASE OF THE TAFII250 FAMILY 2 (HAF2), a member of the TAF1 family, influences histone acetylation and expression of the light‐responsive genes *RBCS* and *CAB2* (Bertrand *et al*., 2005). Interestingly, histone acetylation has been associated with UVR8‐dependent transcriptional regulation (Cloix & Jenkins, 2008; Velanis *et al*., 2016). In particular, chromatin immunoprecipitation of seedlings undergoing UV‐B exposure revealed an enrichment for H3K9K14 acetylation at UVR8 regulated genes (Velanis *et al*., 2016).

The Arabidopsis genome encodes for at least 18 HDACs that are classified in three main classes: the RPD3/HDA1 large family, based on the homology to the *S. cerevisiae* RPD3 complex; the NAD‐dependent Sirtuins (SRTs) and the plant‐specific HD2 family (Pandey *et al*., 2002). HD1/HDA19 has been the first reported example of HDAC impacting light‐induced gene expression and reduced H3K9 acetylation levels at *RBCS, CAB2* and *LHCB1*, as well as defining *PHYA* expression during dark‐to‐light transitions (Benhamed *et al*., 2006; Jang *et al*., 2011). Recent work also has shown that the HDA19 and SIN3‐like (SNLs) function as negative regulators of de‐etiolation (Jing *et al*., 2021). Indeed, loss‐of‐function of *HDA19* or different *snl* mutants show defective hypocotyl elongation. The SNL complex can directly interact with HY5, as well as deacetylate its locus together with *B‐BOX CONTAINING PROTEIN 22* (*BBX22;* Jing *et al*., 2021). Altogether, the study by (Jing *et al*., 2021) suggests that light triggers HY5‐dependent recruitment of the HDA19 complex to promote selective deacetylation and subsequent transcriptional repression of target genes. HDA15 operates through a similar mechanism by interacting with HY5 to negatively regulate hypocotyl elongation under R and FR light (Zhao *et al*., 2019). Furthermore, genome‐wide studies revealed that HDA15 and HY5 are required for repressing a subset of cell wall and auxin biosynthesis genes (Zhao *et al*., 2019). In addition, HDA15 interacts directly with PIF3 (Kim *et al*., 2003), promoting histone hypoacetylation and repress transcription in the dark (Liu *et al*., 2013). Likewise to HY5, PIF1 together with HDA15 contributes to the downregulation of light‐responsive genes to prevent seed germination under dark conditions (Gu *et al*., 2017). Interestingly, the nuclear abundance of chromatin modifiers is regulated not only at the transcriptional level or by ubiquitin‐mediated protein degradation, but also by HDA15 nucleo‐cytoplasmic partitioning (Alinsug *et al*., 2012) impacting on global histone acetylation levels (Liu *et al*., 2013).

Recently, HDA6 was shown to reduce H3K27ac levels on the *ABI5* (*ABSCISIC ACID INSENSITIVE 5*) promoter as a means of regulating seedling establishment downstream of light and hormone stimuli (D. Xu *et al*., 2020), whereas HDA9 and HDA15 modulate transcription at the crosstalk between light and temperature (Van Der Woude *et al*., 2019; Y. Shen *et al*., 2019; Yang *et al*., 2020a). HDA9 has been shown to control hypocotyl elongation in response to warm ambient temperatures and inhibit the transcription of autophagy‐related genes (*ATGs*) in a HY5‐dependent manner by deacetylating *ATG5* and *ATG8e* loci (Yang *et al*., 2020a). Such inhibition is reduced in darkness where HY5 is targeted for degradation via the 26S proteasome, thereby dissociating HDA9 from *ATG* loci.

### 3. Histone variants

In plants as in other organisms, the histone variant H2A.Z can replace the canonical H2A variant to modulate gene expression in response to environmental changes (Kumar & Wigge, 2010; Bieluszewski *et al*., 2022). In Arabidopsis, H2A.Z incorporation is notably mediated by the chromatin‐remodeling factor INO80 to repress the expression of light‐related genes, including *HY5* and *HYH* (*ELONGATED HYPOCOTYL 5‐HOMOLOG*) by modulating nucleosome density (Yang *et al*., 2020b).

**Fig. 2:**
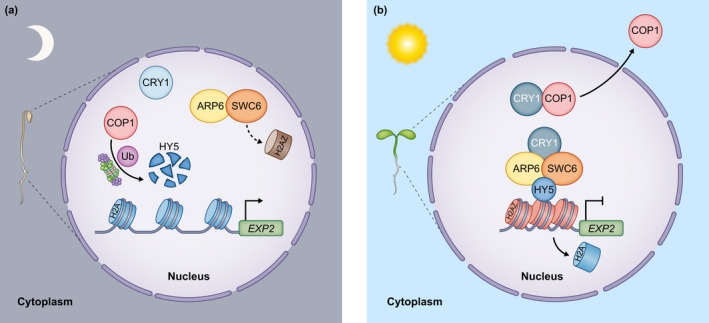
CRY1 contributes to H2A.Z deposition during photomorphogenesis in *Arabidopsis thaliana*. (a) In the dark, CRY1 is inactive and does not interact with either COP1 or the SWR1 complex components ARP6 and SWC6. As a result, COP1 targets HY5 for ubiquitination followed by degradation. The absence of HY5 limits the recruitment and H2A.Z deposition directed by ARP6 and SWC6, over HY5 regulated loci such as *EXPANSIN2* (*EXP2*), which in turn remains transcriptionally active leading to enhanced hypocotyl elongation (skotomorphogenesis). (b) Upon blue light illumination, CRY1 becomes activated and directly interacts with COP1, thereby promoting its translocation from the nucleus to the cytoplasm and allowing HY5 accumulation. In addition, CRY1 together with HY5 stabilizes the SWR1 complex containing ARP6 and SWC6 over HY5 target genes and increases H2A.Z–H2A nucleosome exchange. The expression of *EXP2* and of other positive regulators of cell elongation is therefore reduced, uncovering a novel CRY1‐mediated photomorphogenesis mechanism (Mao *et al*., 2021). CRY1, CRYPTOCHROME 1; COP1, CONSTITUTIVE PHOTOMORPHOGENIC 1; SWR1, SWI2/SNF2‐Related 1 Chromatin Remodeling Complex ARP6, ARP6, ACTIN‐RELATED PROTEIN 6; HY5, ELONGATED HYPOCOTYL 5, EXP2, EXPANSIN 2. Blunt‐ended arrows indicate repression or no transcription.

Photoreceptors are also involved in H2A.Z deposition. Under blue light, CRY1 can physically associate with two subunits of the SWI2/SNF2‐Related 1 Chromatin Remodeling Complex (SWRI‐C), in particular, ACTIN‐RELATED PROTEIN 6 (ARP6) and SWR1 complex subunit 6 (SWC6) that catalyze H2A.Z incorporation into the chromatin (Fig. 2; Table 1). This regulates HY5‐dependent gene expression during de‐etiolation (Mao *et al*., 2021; Fig. 2). In a follow‐up study, the Pfr form of phyB was shown to directly interact with ARP6 and SWC6 (Wei *et al*., 2021). Interestingly, this interaction was required to promote H2A.Z deposition specifically on the *YUCCA9* locus during de‐etiolation. Unlike the previous study, this association was only partially dependent on HY5. Further evidence demonstrated that the H2A.Z removal from shade‐induced loci such as *ATHB2* (*ARABIDOPSIS THALIANA HOMEOBOX PROTEIN 2*) depends on PIF7 association with their promoters. PIF7 can directly interact with the EEN subunit of the INO80‐complex and thereby modulates H2A.Z deposition on key loci. Interestingly, H2A.Z depletion precedes induction of gene expression, suggesting that chromatin remodeling anticipates transcriptional activation (Willige *et al*., 2021). Furthermore, PIF7 recruitment to DNA triggers histone hyperacetylation in a light‐quality‐dependent manner (Willige *et al*., 2021). In an independent study, H2A.Z occupancy was further found to be induced by light through an interaction between NF‐YC (NUCLEAR FACTOR‐Y, Subunit C) and the SWRI‐C subunit ARP6 (C. Zhang *et al*., 2021).

Finally, as described above, the stress‐inducible and structurally atypical H.3 linker histone variant is induced under unfavorable light conditions such as low light intensity. Its dynamic incorporation into chromatin, particularly at multiple genes in a euchromatin context, presumably triggers the formation of specific chromatin compaction states to accompany or facilitate transcriptional reprogramming (Rutowicz *et al*., 2015).

## IV. Light‐mediated regulation of chromatin architecture

### 1. Higher‐order chromatin organization

The 3D structure of chromatin and spatial distribution of the genome within the nucleus play a pivotal role in the regulation of the plant transcriptome (Strahl & Allis, 2000). Seminal reports have shown that the plant chromatin landscape changes rapidly in response to environmental stimuli such as light and temperature (Tessadori *et al*., 2009; van Zanten *et al*., 2010a,b, 2012; Bourbousse *et al*., 2015, 2020; Perrella & Kaiserli, 2016; Perrella *et al*., 2020). When Arabidopsis seedlings first emerge from the soil and perceive light, cryptochrome (CRY1 and CRY2) activity allows for the nucleus to increase in size along with the rapid formation of so‐called chromocenters, a direct outcome of the compaction of heterochromatic regions (Bourbousse *et al*., 2015). On the contrary, under dark conditions COP1 and DET1 contribute to sustaining the de‐compacted status of heterochromatin in most cells of etiolated cotyledons (Bourbousse *et al*., 2015). Light not only acts on the 3D organization of pericentromeric and other heterochromatic regions, but also triggers the translocation of several light‐responsive genes from the inner nuclear space to the periphery, before their transcriptional activation (Feng *et al*., 2014). Interestingly, light‐induced gene motion involves the R/FR absorbing phytochromes phyA and phyB, whilst COP1, DET1 and PIFs impede the aforementioned event (Feng *et al*., 2014). Although lacking a genome‐wide perspective on variations of chromatin subnuclear organization, these studies identified COP1 and DET1 as central light signaling components influencing the subnuclear organization of both protein‐coding genes and heterochromatic genome scaffolds. Future studies may help decipher functional and mechanistic interplays between these two regulatory levels and genome expression reprogramming during light‐driven cellular transitions.

### 2. Gene loops

**Fig. 3:**
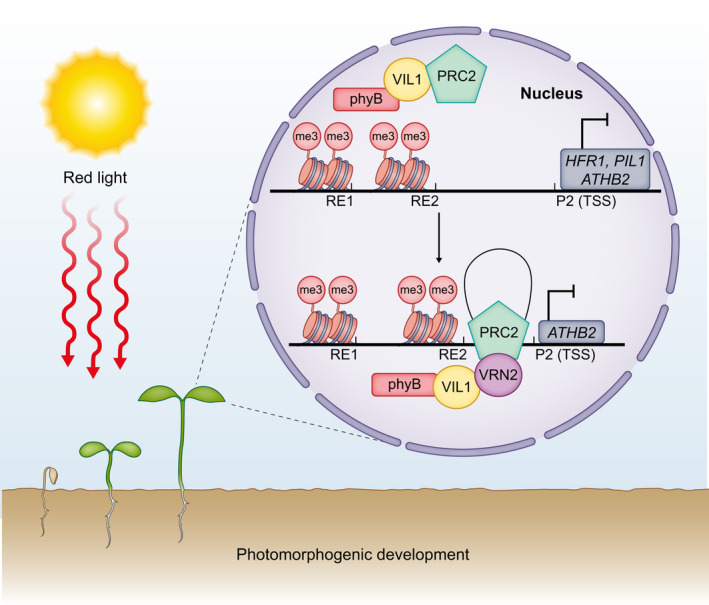
Photo‐activated phyB and PRC2‐associated VIL1 mediate chromatin modifications on hypocotyl elongation marker genes to promote photomorphogenic development. PhyB and VIL1 are essential for the PRC2‐dependent deposition of H3K27me3 at the *HFR1, PIL1* and *ATHB2* loci (Kim *et al*., 2021). PhyB (Pfr) associates with the VIL‐PRC2 module to form a repressive chromatin loop between the RE1 (regulatory element) and P2 promoter region upstream of the *ATHB2* transcriptional start site (TSS) to inhibit *ATHB2* expression (Kim *et al*., 2021). PhyB, PHYTOCHROME B; PRC2, Polycomb Repressive Complex 2; VIL1, VERNALIZATION INSENSITIVE 3‐LIKE 1; HFR1, LONG HYPOCOTYL IN FAR‐RED; PIL1, PHYTOCHROME INTERACTING FACTOR 3‐LIKE 1; ATHB2, ARABIDOPSIS THALIANA HOMEOBOX PROTEIN 2; blunt‐ended arrows indicate repression or no transcription.

Chromatin looping is a regulatory mechanism that facilitates interactions between genomic regions regardless of their spatial proximity (Sotelo‐Silveira *et al*., 2018; Dong *et al*., 2020; Gagliardi & Manavella, 2020; Domb *et al*., 2022). Chromatin loops grant regulatory genomic elements access to their targeted intrachromosomal loci and thus these structures actively influence transcription (Miele & Dekker, 2008; Cavalli & Misteli, 2013; Sotelo‐Silveira *et al*., 2018). Recent findings from (Kim *et al*., 2021), demonstrated that the light and temperature sensor phyB works cooperatively with the Polycomb Repressive Complex PRC2‐associated VIL1 (VERNALIZATION INSENSITIVE 3‐LIKE1) to induce the formation of a repressive chromatin loop over the *ATHB2* gene (Fig. 3; Kim *et al*., 2021). VIL1, a member of the VERNALIZATION INSENSITIVE 3 (VIN3) family of proteins, is a PLANT HOMEODOMAIN (PHD) finger protein that mediates the initiation of flowering by repressing the expression of *FLOWERING LOCUS C* (*FLC*) in a PcG (Polycomb group)‐dependent fashion (Sung *et al*., 2006; Kim & Sung, 2013). VIL1 and phyB repress the expression of three hypocotyl marker genes, *ATHB2*, *HFR1* (*LONG HYPOCOTYL IN FAR‐RED*) and *PIL1* (*PHYTOCHROME INTERACTING FACTOR 3‐LIKE 1*) through PRC2‐dependent deposition of the H3K27me3 repressive mark upstream of their TSS (Kim *et al*., 2021). Interestingly, *vil1‐1 phyB‐9* double mutant seedlings demonstrated elongated hypocotyl phenotypes correlating with the degree of *ATHB2* upregulation (Kim *et al*., 2021). Furthermore, to fully inactivate *ATHB2* expression, phyB and the PRC2‐VIL1 complex form a repressive gene loop between its RE1 regulatory element and TSS regions (Kim *et al*., 2021). This chromatin loop is contingent on the physical interaction of photo‐activated phyB and VIL1, rendering the formation of this regulatory structure R‐light dependent (Fig. 3; Kim *et al*., 2021).

### 3. R‐loops

R‐loops constitute a specialized class of chromatin loop structures that regulates gene expression. These nucleic acid structures consist of three strands, a DNA : RNA hybrid and a displaced single‐stranded DNA (ssDNA) molecule (Thomas *et al*., 1976; White & Hogness, 1977). R‐loops are a common element amongst eukaryotic and prokaryotic genomes that occurs naturally during vital cellular events such as transcription and epigenetic modifications (Skourti‐Stathaki & Proudfoot, 2014; Santos‐Pereira & Aguilera, 2015; Chédin, 2016; Gaillard & Aguilera, 2016; Niehrs & Luke, 2020). Disruption in R‐loop homeostasis confers genome instability and DNA damage through the induction of transcription‐replication conflicts (TRCs) and hindrance of DNA repair processes (Helmrich *et al*., 2011; D'Alessandro *et al*., 2018; Lu *et al*., 2018; Rinaldi *et al*., 2021). Simultaneously, programmed site‐specific R‐loop formation is important for the mitigation of UV‐induced DNA lesions by signaling the alteration of global spliceosome dynamics, which highlights the pleiotropic effect of R‐loops on genome integrity (Tresini *et al*., 2015). Although the functional role of R‐loops has long been investigated mainly in mammalian models, research in plants is catching up, as R‐loops have been recognized as an important mechanism in gene regulation and a potentially valuable tool in agriculture‐oriented applications. R‐loops have also been shown to indirectly affect epigenetic signatures, through the action of noncoding RNA‐generated loop formation. Long noncoding RNAs (lncRNAs) physically associate with proteins, DNA and RNA, whilst they also can invade double‐stranded DNA to form R‐loops (Statello *et al*., 2021). R‐loops play a key role in polar auxin transport, root development, regulation of flowering time, and RNA splicing, whilst also contributing to genome instability of the chloroplasts and nucleus (Sun *et al*., 2013; Conn *et al*., 2017; Shafiq *et al*., 2017; Yang *et al*., 2017; Yuan *et al*., 2019), however, there are limited studies dissecting how R‐loop patterns are affected by light. A recent report showed that Arabidopsis R‐loop dynamics remain almost invariable in response to diverse light conditions (W. Xu *et al*., 2020). Interestingly, there was a striking difference in sense R‐loop formation in plants exposed to light vs those grown in the dark (W. Xu *et al*., 2020), which could imply a potential role for R‐loops in plant physiological responses to exogenous stimuli such as the transition to photomorphogenic growth.

In plants, R‐loops are highly prone to form around promoter regions and gene bodies, however, contrary to mammals they are less enriched at terminator sites (Xu *et al*., 2017). Intriguingly, R‐loop formation is associated with transcriptionally‐permissive histone marks including H3K9Ac, H3K36me3 and H3K4me3/me2, whilst in genomic regions enriched in heterochromatin‐related epigenetic marks R‐loop localization is significantly lower (Xu *et al*., 2017). In Arabidopsis, there is strong evidence that R‐loops are involved in RdDM‐mediated (RNA‐directed DNA methylation) gene silencing, as indicated by the strong presence of R‐loop formation in Pol IV‐transcribed noncoding sites (Xu *et al*., 2017). In rice, R‐loop identification by Fang *et al*. (2019) suggested that R‐loops and chromatin marks are intrinsically linked on a genome‐wide scale because DNA methylation as well as several histone marks such as H3K9me2, H3K4me3 and H3ac, can enhance R‐loop formation (Fang *et al*., 2019). Furthermore, whilst RNA methylation (R‐m^6^A) positively affects R‐loop formation as well as gene expression (described in the section ‘Light‐driven regulation of the epitranscriptome’), DNA m^6^A methylation can potentially have a negative impact on transcription when accompanied by R‐m^6^A (P. Zhang *et al*., 2021).

The role and molecular mechanism of R‐loop formation in regulating gene expression in response to changes in light quality, quantity and duration is still largely unexplored. Identifying the key components stabilizing or promoting the formation of 3D chromatin structures and characterizing whether these components are regulated by light or interact with photoreceptors and light signaling factors promises to expand our knowledge on nuclear processes contributing to plant adaptation to light. Deciphering how the 3D chromatin organization contributes to the genetic plasticity of plants in addition to how the spatial distribution of the genome changes in response to light cues will deepen our knowledge of functional genomics and enhance efforts for the improvement of future agricultural practices.

## V. Light‐driven regulation of the epitranscriptome

In plants, m^6^A and m^5^C are the most prevalent mRNA modifications. As for chromatin modifications, RNA methylation is deposited by ‘writers’ (RNA methyltransferases), removed by ‘erasers’ (RNA demethylases) and recognized by ‘readers’ (Liang *et al*., 2020). Two m^6^A writers have been identified in plants, a methyltransferase complex composed of at least five proteins, namely mRNA ADENOSINE METHYLASE (MTA; ortholog of METTL3 in animals), METHYLTRANSFERASE B (MTB; ortholog of METTL14), FKBP12 INTERACTING PROTEIN 37 (FIP37; ortholog of WTAP), VIRILIZER (ortholog of WIRMA) and HAKAI (Yue *et al*., 2019), that is responsible for the majority of mRNA methylation, and FIONA1 that deposits m^6^A at U6 snRNAs and at a subset of mRNAs (Sun *et al*., 2022; Wang *et al*., 2022; Xu *et al*., 2022). Both writers have been associated with plant light signaling and light regulation of circadian clock entrainment (Kim *et al*., 2008; Parker *et al*., 2020; X. Wang *et al*., 2021).

A first hint that RNA methylation could play an important role in plant light responses was provided by the analysis of the m^6^A epitranscriptome in two *A. thaliana* natural accessions collected at locations where annual PAR is at the two extremes of the natural range (*Can‐0* from the Canary Islands and *Hen‐16* from Sweden; Luo *et al*., 2014). Although m^6^A patterns were found to be generally conserved across the two accessions, with > 5000 genes showing enrichment around the start and stop codons and 3′ UTRs, the *Can‐0* accession had overall higher m^6^A levels and higher number of marked transcripts than *Hen‐16*. Strikingly, more than half of the methylated transcripts encode proteins with a chloroplastic function in both lines (Luo *et al*., 2014). Functional analyses are required to assess if this feature confers advantageous traits under different PAR environments. Furthermore, the major mRNA m^5^C methyltransferase in rice, OsNSUN2, was found to play an essential role in chloroplast heat acclimation. Its Arabidopsis ortholog *TRM4B* selectively methylates the transcripts of genes involved in photosynthesis, chloroplast development and detoxification to regulate their translation and preserve chloroplast homeostasis (Tang *et al*., 2020).

Recent studies have shown that the blue light receptors CRY1 and CRY2 were found to physically associate with MTA, MTB and FIP37 (X. Wang *et al*., 2021). Many messenger RNAs of *cry1cry2* mutant plants show a massive decrease in m^6^A modification, especially over 3′ UTRs. Upon exposure to blue light, the CRY2–MTA complex undergoes rapid condensation into photobodies, suggesting that concentrating the m^6^A MTA/MTB/FIP37 writer may facilitate mRNA methylation in response to light. Cryptochrome‐mediated RNA methylation regulates transcript stability of many genes including *PHYA*, *PHOT2* and *UVR8* photoreceptors and the 10 central circadian oscillator genes, thereby providing a new mechanism by which light regulates the clock (X. Wang *et al*., 2021).

With regards to the second m^6^A writer, FIONA1, was first identified by a causative mutation in an Arabidopsis EMS genetic screen for early flowering (Kim *et al*., 2008), but its molecular function as an RNA methyltransferase has emerged only recently (Sun *et al*., 2022; Wang *et al*., 2022; Xu *et al*., 2022). In this seminal study, FIONA1 was reported to extend the period length of the expression of central oscillator genes including *CIRCADIAN CLOCK ASSOCIATED 1* (*CCA1*), *LATE ELONGATED HYPOCOTYL* (*LHY*) and *TOC1*, and to increase mRNA levels of key flowering regulatory genes *CONSTANS* (*CO*) and *FLOWERING LOCUS T* (*FT*; Kim *et al*., 2008). *CCA1* and *LHY* transcripts have reduced m^6^A levels in a *fiona1* mutant, suggesting that FIONA1 could target central oscillator transcripts to regulate their periodicity. Two studies using methylated RNA immunoprecipitation (meRIP‐seq) identified approximately 1000 genes with hypomethylated transcripts in *fiona1* mutant plants, which predominantly lacks m^6^A in their 3′ UTR (Sun *et al*., 2022; Wang *et al*., 2022), whereas direct RNA sequencing in a knock‐down *FIONA1* mutant line identified > 2000 transcripts preferentially hypomethylated before the stop codon (Xu *et al*., 2022). It is not clear whether the discrepancy between these different studies originates from genetic, technological or analytical differences. In addition, because FIONA1 was found to directly target *CRY2* transcripts and dampens their level (Wang *et al*., 2022), it is difficult to disentangle direct effects of *FIONA1* loss‐of‐function from indirect effects due to the perturbation of the *CRY2*‐MTA RNA methyltransferase complex. Targeted analyses through RNA immunoprecipitation identified FIONA1 physical association with transcripts from four additional genes in addition to *CRY2*: *FLC*, *SUPPRESSOR OF OVEREXPRESSION OF CONSTANS 1* (*SOC1;* Xu *et al*., 2022) and the associated transcriptional activator *CO*, as well as the transcription factor *PIF4* (Wang *et al*., 2022). FIONA1 methylation of *PIF4* transcripts decreases their stability and was proposed to participate in R/FR light phytochrome signaling (Wang *et al*., 2022). Indeed, in addition to clock‐related phenotypes, *fiona1* mutants display de‐etiolation phenotypes under continuous R and FR light but not under white light or darkness. Altogether, these findings suggest that FIONA1‐directed m^6^A deposition positively regulates photomorphogenesis downstream of phytochrome signaling.

Interestingly, the function of RNA methylation in circadian clock entrainment by light appears to be evolutionarily conserved from plants to metazoans (Fustin *et al*., 2013). The N6‐methyladenosine level peaks during the night in the seagrasses *Cymodocea nodosa* and *Zostera marina* (Ruocco *et al*., 2020), and likewise m^6^A levels increase at night in mice liver cells (Wang *et al*., 2015), suggesting that circadian oscillation of the epitranscriptome could be a conserved feature across kingdoms, yet this remains to be assessed in Arabidopsis. Similar to plants, deficiency in CRY‐dependent blue light perception in mammals decreases m^6^A transcript levels (Wang *et al*., 2015) and human CRY2 can interact with the m^6^A writer complex subunits METTL3, METTL14 and WTAP (X. Wang *et al*., 2021), suggesting the existence of conserved mechanisms connecting light signaling to epitranscriptomic regulations.

## VI. An epigenetic perspective on light regulation of genome and epigenome dynamics

After the discovery that damaging doses of UV‐B prevalently affect methylated cytosines (Willing *et al*., 2016), much of the studies exploring the link between light and the DNA methylome have focused on UV. A mechanistic connection between UV‐B signaling and DNA methylation has recently been unraveled by Jiang *et al*. (2021). Arabidopsis plants grown under UV‐B‐containing light display DNA hypomethylation at thousands of, mainly pericentromeric, TE‐rich regions. Accordingly, silencing of many TEs is altered in UV‐B‐grown plants, which supports the early observation in maize of UV‐B‐induced expression and transposition of the Mutator (Mu) DNA transposon (Walbot, 1999; Questa *et al*., 2013). UV‐C radiation, which is extremely harmful but almost completely absorbed by the atmosphere, has also been shown to trigger DNA methylation changes in heterochromatin, and alter epigenome integrity in plants defective in any of the photodamage repair pathways (Graindorge *et al*., 2019). Specifically, recognition of the lesions by DNA DAMAGE‐BINDING PROTEIN 2 (DDB2) followed by Global Genome Repair (GGR) or by small RNA‐mediated GGR, a related pathway triggered by the production of UV‐induced siRNA (uviRNAs) at photodamaged regions (Schalk *et al*., 2017), prevent gain of DNA methylation, whereas direct repair by the photolyases prevents loss of DNA methylation (Graindorge *et al*., 2019). A recent report has shown that the MED17 requirement for small noncoding RNA biogenesis and heterochromatic loci repression also plays a role in DNA damage repair in response to UV‐B irradiation in Arabidopsis (Giustozzi *et al*., 2022).

Unexpectedly, the genomic loci undergoing differential methylation in response to UV‐B and UV‐C are largely distinct, suggesting that effects of UV‐B on the epigenome are independent of DNA damage (Jiang *et al*., 2021). Supporting this observation, UV‐B‐dependent loss of DNA methylation and transcriptional de‐repression of TEs depends on signaling through the UV‐B photoreceptor UVR8. It is noteworthy that the UV‐B‐dependent DNA methylation landscape largely overlaps with targets of the DRM2 DNA methyltransferase. To provide a link between UV‐B perception and DNA methylation, a physical interaction between UVR8 and the ubiquitin‐associated (UBA) domain of DRM2 was found to impede DRM2 activity *in vitro* and chromatin binding *in planta*. UV‐B converts cytosolic UVR8 homodimers into active nuclear monomers capable of interacting with DRM2 to inhibit its activity, leading to DNA hypomethylation (Jiang *et al*., 2021). Several hypotheses about the functional relevance of UV‐chromatin mechanisms in stress acclimation, stress memory through priming or across generations, and the evolution of genetic diversity can be envisioned, as described below.

With regard to stress acclimation, UV‐B‐triggered DNA hypomethylation could favor UV tolerance by influencing gene expression. For instance, in maize, *P1* (*PERICARP COLOR1*) encodes a R2R3‐MYB transcription factor that promotes the accumulation of UV‐protective flavonoids. Increased *P1* expression in leaves of high‐altitude landraces and in response to UV‐B treatments is caused by loss of DNA methylation along its promoter and coding sequences (Rius *et al*., 2016). Whether the regulation of methylation at the *P1* locus relies on maize homologs of UVR8 and DRMs remains to be addressed.

Because DNA methylation is metastable and can be inherited through mitosis (Law & Jacobsen, 2010), modulation of the DNA methylation landscape has long been proposed to constitute a memory mechanism enabling the plant to better respond to subsequent environmental cues. Such a priming mechanism has been unveiled for UV‐B stress in Arabidopsis, where a single, short and nondamaging UV‐B treatment stimulates resistance against re‐exposure after three days (Xiong *et al*., 2021). Although the priming mechanism has been shown to rely on UV‐B photoperception by UVR8, potential variations in the DNA methylation status and impact on gene expression have not yet been assessed. Interestingly, priming of Arabidopsis responses to stress also has been established for excess light, in which recurrent exposure improves photosynthesis in new and old leaves (Crisp *et al*., 2017; Ganguly *et al*., 2018), suggesting an epigenetic transmission from the exposed meristematic cells to new organs or the existence of a mobile signal from exposed to nonexposed cells. Yet, no significant DNA methylation changes could be observed between primed and unprimed plants, and mutants affected in DNA methylation deposition, maintenance or removal, displayed no priming defects in this study (Ganguly *et al*., 2019). The latter observation suggests the existence of a light priming mechanism independent of DNA methylation, potentially controlled by other chromatin processes or unidentified regulatory mechanisms.

Long‐term, transgenerational, memory of UV‐B exposure has also been unveiled in the clonal plant *Glechoma longituba* (ground ivy), in which parental ramets exposed to UV‐B produce offspring ramets manifesting an ‘escape strategy’ when foraging in a UV heterogeneous environment, whereas ramets from ‘naïve’ parents do not show any behavioral preference (Quan *et al*., 2021). In this study, UV‐B stress reduced overall DNA methylation level in parental ramets, a hypomethylation event that appears to be maintained in offspring ramets. At this stage, existence of an epigenetic memory controlling foraging behavior remains to be established.

It is tempting to speculate that DNA hypomethylation induced by UV‐B may increase the evolutionary potential of plant populations by enhancing TE mobilization and reducing genome stability. Capacity of TE mobilization to rapidly increasing Arabidopsis genetic and phenotypic diversity recently has been established, a process that further allows the selection of individuals better adapted to new environments in the offspring (Baduel *et al*., 2021). Remarkably, UV‐B induced hypomethylation at thousands of genomic regions of the tropical mangrove *Rhizophora apiculata* is associated with the reactivation of a large population of TEs which sometimes are positioned adjacent to UV‐B inducible genes (Y. Wang *et al*., 2021). These observations may indicate that new TE insertions have been co‐opted by the plant genome to enhance fitness in response to UV.

## VII. Conclusions and future directions

Plant responses to diverse and fluctuating light regimes are governed by changes in gene expression. Here, we focus on how light triggers changes in plant chromatin structure and nuclear architecture to coordinate plant adaptation and development. Our knowledge of the role of chromatin secondary and tertiary structures through looping as well as protein and nucleic acid modifications in modulating photoregulated transcripts is growing rapidly. In particular, transcriptional regulators including chromatin remodelers, histone variants and scaffold proteins are being discovered or assigned functions related to environmental signal integration.

Furthermore, there is increasing evidence for the prominent role of biomolecular condensates in compartmentalizing light signaling processes and facilitating nuclear signal integration in a fast, energy‐efficient and reversible manner. The majority of plant photoreceptors, with the exception of phototropins, operate in the nucleus (Perrella & Kaiserli, 2016). Therefore, nuclear signal integration is key for optimal transcriptional regulation of light‐responsive genes. The formation of biomolecular condensates is an emerging regulatory process in plant photobiology. Reversible, light‐induced formation of nuclear bodies, also referred to as photobodies, has been known for decades (Van Buskirk *et al*., 2012; Pardi & Nusinow, 2021) and potentially promote protein–nucleic acid crosstalk and therefore environmental signal integration within the nucleus. However, only recently nuclear bodies were shown to aggregate CRY2 (X. Wang *et al*., 2021) or ELF3 (Jung *et al*., 2020) through liquid–liquid phase separation (LLPS), a reversible process based on mixing and unmixing of a dense and diluted liquid phase. Biomolecular condensation regulates the compartmentalization of molecular processes at the subcellular and subnuclear level, and plays a key role in mediating reversible stress and adaptive responses to endogenous and environmental stimuli. Intrinsically Disordered protein Regions (IDRs), such as those found in ELF3, and RNAs, have been shown to promote the formation of nuclear condensates through LLPS (Salladini *et al*., 2020; Roden & Gladfelter, 2021). In the case of CRY2, blue light triggers the formation of spherical, reversible and highly dynamic nuclear bodies that co‐condense with m6A methyltransferases through LLPS (X. Wang *et al*., 2021). As a result, a novel CRY2 function was discovered in regulating m^6^A writer activity through a CR‐dependent and blue‐light mediated LLPS recruitment mechanism that results in controlling 10% of the Arabidopsis mRNA abundance through methylation (X. Wang *et al*., 2021). A recent report showed that phyB photobodies also form through LLPS (Chen *et al*., 2022). More specifically, phyB self‐associates into liquid‐like droplets through its C‐terminus in response to R light, whereas the intrinsically disordered N‐terminal extension modulates phyB phase separation in response to temperature changes (Chen *et al*., 2022). However, further evidence is essential to determine if phyB can intrinsically form biomolecular condensates in an *in vitro* system.

Whether all light‐induced nuclear foci form through LLPS remains to be established. There is strong indication that post‐translational modifications (including SUMOylation and phosphorylation) as well as association with RNA, histones and scaffold proteins facilitate the formation of biomolecular condensates. In future studies, the advancement of bioimaging and genome‐enabled experimental tools such as fluorescence in situ hybridization (FISH), Chromatin Conformation Capture (4C and Hi‐C) and related techniques enabling us to reach a 3D perspective in DNA and protein networks (Grob, 2020; Zhang & Wang, 2021), and their integration in 3D‐Genomics approaches, should revolutionize our understanding of how chromatin architecture dynamics set the ground for genome regulation in response to light signals. The molecular and biological significance of light‐triggered compartmentalization in the nucleus is anticipated to be multifaceted as photobodies are sites of diverse processes and hubs of signaling networks. Therefore, light‐reversible formation of nuclear domains regulating adaptive responses at the chromatin, transcriptional and post‐transcriptional levels may confer an ultimate rheostat modulating plant adaptive responses to fluctuating environmental conditions and a potential target for agriculture. Yet, it is still unclear what the function of light‐induced nuclear foci is, and whether their formation is involved in promoting signaling or desensitization. Highly sensitive imaging, proteomic and next generation sequencing strategies are now available in order to dissect the molecular processes and components of chromatin hubs in distinct cell‐type‐specific contexts. Future studies aimed at characterizing the molecular mechanisms and physiological significance of light‐responsive chromatin regulatory complexes will undoubtedly provide potential targets for fine‐tuning plant growth and adaptation in response to a changing environment. Although photoreceptors are the obvious candidates for genetic manipulation, their effect on plant development is pleiotropic, and therefore modulating their function could be detrimental in both agricultural and natural contexts. The chromatin and nuclear landscape provide a tunable switch for promoting adaptation without compromising growth, which is the ultimate strategy for epi‐breeding.

## Author contributions

All authors (FB, CB, EK, EP, GP, GS, AZ) contributed to the conceptualization and writing of the review.
